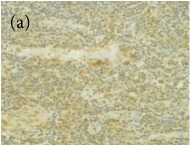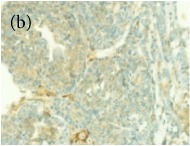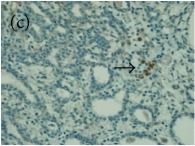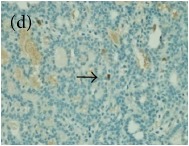# Oral Administration of Tualang and Manuka Honeys Modulates Breast Cancer Progression in Sprague-Dawley Rats Model

**DOI:** 10.1155/2017/5904361

**Published:** 2017-04-05

**Authors:** Sarfraz Ahmed, Siti Amrah Sulaiman, Nor Hayati Othman

**Affiliations:** ^1^Department of Pathology, School of Medical Sciences, Universiti Sains Malaysia, Kubang Kerian, 16150 Kelantan, Malaysia; ^2^Department of Biochemistry, Bahauddin Zakariya University, Multan 60800, Pakistan; ^3^Department of Pharmacology, School of Medical Sciences, Universiti Sains Malaysia, Kubang Kerian, 16150 Kelantan, Malaysia

## Abstract

Breast cancer has been recognized as the leading cause of death in women worldwide. Research has shown the importance of complementary and alternative therapies in cancer. In this study, we investigated the antitumoural therapeutic effects of Malaysian Tualang honey (TH) and Australian/New Zealand Manuka honey (MH) against breast cancer in rats. Thirty syngeneic virgin female Sprague-Dawley (SD) rats were induced by the carcinogen 1-methyl-1-nitrosourea (MNU) 80 mg/kg. The treatment started when first palpable tumour reached 10–12 mm in size by dividing rats into following groups: Group 0 (negative control); Group 1 (positive control); and Groups 2 and 3 which received 1.0 g/kg body weight/day of TH and MH, respectively, for 120 days. The data demonstrate that cancer masses in TH and MH treated groups showed a lower median tumour size, weight, and multiplicity compared with the nontreated positive control (*p* < 0.05). Treatment also showed a dramatic slower growth rate (up to 70.82%) compared with the nontreated control (0%) (*p* < 0.05). The antitumoural effect was mediated through modulation of tumour growth, tumour grading, estrogenic activity, and haematological parameters. Our findings demonstrate that systemic administration of TH and MH increases the susceptibility of expression of proapoptotic proteins (Apaf-1, Caspase-9, IFN-*γ*, IFNGR1, and p53) and decreases the expression of antiapoptotic proteins (TNF-*α*, COX-2, and Bcl-xL 1) in its mechanism of action. This highlights a potential novel role for TH and MH in alleviating breast cancer.

## 1. Introduction

Breast cancer has been recognized as the second most common cancer after lung cancer. It is considered as the fifth most common cause of death through cancer and the leading cause of death in women worldwide, surpassing the cervical cancer [1]. In Malaysia, a recent report shows breast cancer as the most frequently diagnosed cancer and the International Agency for Research in Cancer (GLOBOCAN) estimated the ASR (age-standardized rate) of breast cancer in Malaysia as 38.7 per 100,000, listing breast cancer at top among general population of Malaysia [2]. Cancer-therapeutic drugs had an obstacle of collateral damage to the normal cells and tissues [3]. The relapse after chemotherapy, second primary tumours, and resistance to chemotherapeutic drugs have also been reported in breast cancer patients [4]. The alternative remedy to these unavoidable side effects is the use of natural products [5].

Honey has clinched the attention of researchers as a complementary and alternative treatment in modern medicine [5, 6]. It is mainly composed of various sugars, phenolic acids, flavonoids, enzymes, amino acids, proteins, and miscellaneous compounds [5]. It has also been reported as a natural phytoestrogen [7]. Tualang honey (TH) is a multifloral jungle honey. It is produced by bees* “Apis dorsata”* which build their hives on Tualang trees* (Koompassia excelsa)* in Malaysian tropical rainforests [8, 9]. Research has shown that TH exhibits antimicrobial [10], wound-healing [10], antioxidant [9, 11], anti-inflammatory [9], antidiabetic [8, 12, 13], and anticancer effects [14]. Manuka honey (MH) unlike Tualang honey is a monofloral honey, produced by honey bees from nectars of Manuka bush* (Leptospermum scoparium)* throughout New Zealand and Australia [15]. Published literature on Manuka honey indicates its numerous therapeutic properties against several ailments [16]. Several studies reported that honey, being rich in flavonoids and polyphenols, has antiproliferative and antitumoural effects in vitro and in vivo [17]. However, the mechanisms for the anticancer effects of honey need to be fully understood.

Full blood count is a prerequisite investigation in cancer patients and poor blood parameters affect the outcome of malignancies and functioning of the immune system [18]. It has also been reported that prolonged exposure to serum estradiol (E2) is an important risk factor for the promotion of breast cancer. The level of estrogen in breast cancer patients has been a controversy. But, a very recent study shows a higher serum level of endogenous estrogen (E2) and estrogen receptors (ESR1, ESR2) in breast cancer patients with worse prognosis. Antiestrogen therapy in breast cancer patients has been shown to be very effective in preventing the disease and recurrence [19].

Apoptosis is recognized as the principle mechanism of drugs therapy-induced regression in breast cancer [5]. The expression of pro- and antiapoptotic proteins is considered as a hallmark for prognosis of this disease, survival to death [5, 17]. Research has shown that cancer cell lines and pretreated primary tumour samples exhibit low level of expression of proapoptotic and higher expression of antiapoptotic proteins of intrinsic pathway [17]. Apoptotic protease activating factor-1 (Apaf-1), of Caspase-9 apoptotic pathway, is a tumor suppressor gene [20]. Loss of Apaf-1 expression can aid tumour cells in evading immune attack-induced death and/or Caspase-9 mediated apoptosis, resulting in metastasis [20]. Research has suggested that interferon gamma (IFN-*γ*), an immune potentiating agent, favours activation of mitochondrial-operated apoptotic pathway in breast cancer cell lines by interacting with its receptor, interferon gamma receptor 1 (IFNGR1) [21]. Similarly, the crucial role of p53 as a mediator and cytokine of stress and apoptosis in various cell types has been demonstrated. Its contribution to breast cancer remains unclear. It exhibits tumour suppression as well as an oncogenic action [22]. The higher expression of cytokines, tumour necrosis factor alpha (TNF-*α*), cyclooxygenase-2* (COX-2)*, and B-cell lymphoma extra large (Bcl-xL) in inflammatory breast carcinoma was found to be associated with increasing tumour grade and the metastatic behavior of breast carcinomas [23–25].

An investigation on the effects of honey on haematological parameters and cytokines or markers of estrogenic as well as apoptotic mediated pathways has not been undertaken well. The potential “therapeutic” effects of TH and MH in our study were to investigate the antitumoural effects, the histological features, and the tumour grades evaluation. This study also highlights a potential therapeutic role of TH and MH to modulate haematological parameters and the expression of Apaf-1, Caspase-9, IFN-*γ*, IFNGR1, p53, E2, ESR1, TNF-*α*, COX-2, and Bcl-xL in in vivo breast cancer model. To our knowledge, this is the first study to report the modification of haematological parameters and estrogenic activity by administering honey as a potential therapeutic measure in in vivo breast cancer model.

## 2. Materials and Methods

### 2.1. Animals

The experimental protocol used in this study was approved by the animal ethics committee of Universiti Sains Malaysia, Malaysia [USM/Animal Ethics Approval/2011/(68) (306)]. Sprague-Dawley (SD) female rats aged between 28 and 33 days were obtained from Animal Research and Service Centre (ARASC), Universiti Sains Malaysia (USM).

### 2.2. Source of Honey

Tualang honey was supplied by Federal Agricultural Marketing Authority (FAMA), Ministry of Agriculture and Agrobased Industry, Malaysia. The honey samples were filtrated, evaporated at 40°C (to achieve 20% water content), and were subjected to gamma irradiation at 25 kGy for sterilization by STERILE GAMMA™, Selangor, Malaysia. Manuka honey was purchased from the market (packed under license number 1003 for Vitaco Health (NZ) Ltd., New Zealand and imported and distributed by Cambert (M) Sdn.Bhd, Malaysia).

### 2.3. Study Design

For tumour induction, the carcinogen MNU (Catalog number N1517-1G, Sigma, USA) was dissolved in 0.9% NaCl solution acidified to pH = 5.0 with 0.05% acetic acid by gentle heating up with hot tap water accompanied by vigorous shaking [26]. This model was developed as described by [26]. MNU was injected intraperitoneally as per 80 mg/kg body weight of the rats at the age of 40 days. A total of 40 female SD rats were divided into 4 groups with 10 animals in each group. These rats were housed in a standard cage with commercial pine chip bedding in a well ventilated animal room with a 12 h day/night cycle, maintained on standard and balanced rat feed diet, and had free access to water ad libitum. The rats were acclimatized to the animal room conditions for at least one week prior to the experimentation. Honey treatment by oral feeding was started when first palpable tumour reached 10–12 mm in size. The treatment was planned to be continued till day 120th. The grouping of the rats was as follows:

- Group 0: negative control; no tumour induction and no honey treatment (normal rats)
- Group 1: positive control; rats bearing breast cancer, but no honey treatment
- Group 2: breast cancer bearing rats treated with TH 1.0 g/kg body weight/day
- Group 3: breast cancer bearing rats treated with MH 1.0 g/kg body weight/day

The rat breast tissue areas were palpated twice weekly to detect the appearance of cancer masses and to monitor their progression. The number, size, and positions of the tumours were recorded on palpation. Tumours were measured in length and width weekly to calculate the size. The tumor size progression (tumour size = 1/2 (length × width^2^)) and assessment of reduction in size were measured as described previously [27]. On 120th day of treatment, the rats were sacrificed after i.p injection of pentobarbital 100 mg/kg body weight. The blood samples were collected by cardiac puncture and placed in plain tubes. Blood samples were left to clot for 2 hours prior to centrifugation for 15 minutes at 4000 rpm. Approximately 3 ml of serum was collected and stored at −80°C until assayed. Tumor masses were examined in vivo then excised. Each cancer mass was fixed in neutral buffered formalin for histological and immunohistochemical analysis.

### 2.4. Determination of Full Blood Count (FBC)

FBC was carried out using an automated cell count analyzer (Sysmex KX-21, Japan) by noncyanide haemoglobin analysis. Autoanalyzer was capable to run several parameters for each sample such as haemoglobin concentration, packed cell volume, red blood cell, mean cell volume, mean corpuscular haemoglobin concentration, mean corpuscular haemoglobin, and platelet and white blood cell counts. The equipment sampling probe aspirated 20 *μ*l well mixed blood samples and the result of analysis was obtained accordingly. A total of 8-9 samples were run for FBC for each group.

### 2.5. Histopathological Examination of the Breast Cancer Masses

A total of 103 H & E breast cancer masses were examined and graded, forty-seven masses from positive control group, twenty-three of 1.0 g/kg TH, and thirty-three from 1.0 g/kg MH. The hematoxylin and eosin stained sections were examined under light microscope at 100x, 200x, and 400x magnification using Olympus BX41 microscope (Olympus Optical Co., Ltd., Tokyo, Japan). The stained sections were examined for grading and histological features by a pathologist who was blind to the treatment and control. The cancer masses were graded as per human cancers grading system using the modified Bloom and Richardson method as described by [28].

### 2.6. Determination of Apaf-1, IFN-*γ*, TNF-*α*, and E2 at Serum Level

Seven to eight serum samples per treatment and control groups were analyzed to determine the level of Apaf-1, IFN-*γ*, TNF-*α*, and E2 in 50 *μ*l serum using Apaf-1, IFN-*γ*, TNF-*α*, and E2 ELISA kits (Catalog number BG-RAT10190, Inc., Novatein Biosciences; CSB-E04579r; CSB-E11987r and CSB-E05110r Inc., COSMO BIO, USA, resp.). Standards included serum of known concentrations of Apaf-1, IFN-*γ*, TNF-*α*, and E2 and a serum blank. The ELISA procedure was performed according to the manufacturer's instructions. The results were obtained by calculating the mean absorbance at 450 nm (Spectrophotometer, Thermo Fisher Scientific Inc., Waltham, MA, USA) for each of the duplicate standards, controls, and samples as stated by the manufacturer. A standard curve was created by plotting with the absorbance value as the dependent variable (*Y*-axis) and concentration as the independent variable (*X*-axis); results in an equation formatted are as follows: *y* = *ax*^2^ + *bx* + *c*, with best-fit straight line, where solving for *x* determined the protein concentration of the sample.

### 2.7. Immunohistochemical Analysis for Apaf-1, Caspase-9, p53, FASLG, FADD, IFNGR1, TNF-*α*, COX-2, ESR1, and Bcl-xl in Breast Cancer Masses

A total of 93 cancer tissues, forty from positive control group, twenty-three from 1.0 g/kg TH, and thirty from 1.0 g/kg MH, were immunohistochemically stained for the marker Apaf-1 with mouse monoclonal Anti-Rat Apaf-1 Antigen (Catalog number SC-65891, Inc., Santa Cruz Biotechnology, USA; diluted at 1 : 100), Caspase-9 Rabbit polyclonal Anti-Rat Caspase-9 Antigen (Catalog number GTX73093, Inc., GeneTex, USA; diluted at 1 : 25), FASLG with monoclonal mouse Anti-Rat FASLG Antigen (Catalog number PAB 8018, Inc., Abnova, Taiwan; diluted at 1 : 200), FADD with Rabbit polyclonal Anti-Rat FADD Antigen (Catalog number GTX73104, Inc., GeneTex, USA; diluted at 1 : 25), p53 with monoclonal mouse Anti-Rat p53 Antigen (Catalog number PAB 1801, Inc., Abcam, UK; diluted at 1 : 50), IFNGR1 with Rabbit polyclonal Anti-Rat IFNGR1 Antigen (Catalog number GTX60200, Inc., GeneTex, USA; diluted at 1 : 200),* TNF-α* with polyclonal Rabbit Anti-Rat* TNF-α* Antigen (Catalog number GTX74120, Inc., GeneTex, USA; diluted at 1 : 600), ESR1 with polyclonal Rabbit Anti-Rat ESR1 Antigen (Catalog number PAB 18170, Inc., Abnova, Taiwan; diluted at 1 : 100), COX-2 with polyclonal Rabbit Anti-Rat COX Antigen (Catalog number RB-9072-R7, Inc., Labvision, USA; ready to use), and Bcl-xL with mouse monoclonal Anti-Rat Bcl-xL Antigen (Catalog nunber MS-1334-P1, Inc., Labvision, USA; diluted at 1 : 100). A semiquantitative scoring system developed previously [29] was used to assess the expression of proteins mentioned. The positive stained cells were counted in 10 fields by first author and confirmed by pathologist (NHO) in a blinded manner. The data was presented as a percentage of positivity.

### 2.8. Statistical Analyses

Data were analyzed using IBM SPSS, Statistics version 22. Fisher Exact test was used to analyze the tumour incidence, latency, and grading. Comparisons between mean values of control and treatment groups were analyzed using one-way ANNOVA with post hoc test of Tukey's Honest Significance Differences (Tukey's HSD). A mixed model two-way repeated measures ANOVA was conducted to evaluate the effect of treatments on rats body weight gain and tumour measurements. The time main effect and the experimental groups × time interaction effect were tested using the multivariate criterion of Wilk's lambda (Λ). Comparison of the median values between groups was done by Kruskal-Wallis *H* test followed by Bonferroni's correction. *p* value < 0.05 was considered statistically significant.

## 3. Results

### 3.1. MNU Induction and Its Toxicity

The palpable tumours were seen in experimental Sprague-Dawley rats in all induced groups during postadministration of MNU at dose 80 mg/kg of body weight. Four rats died after 5–7 weeks of post-MNU-induction without apparent sign of toxicity and were replaced to compensate the sample size.

### 3.2. Tumour Multiplicity and % Reduction in Size of Primary Tumours

At the end of study, the rats in positive control which received no honey treatment (Group 1) had the highest median number of tumours (tumour multiplicity) compared with the groups treated with TH and MH (Groups 2 and 3) (*p* > 0.05). The used strengths of TH and MH (Groups 2 and 3) showed a significant % reduction (in size of first three primary tumours developed) and least tumour size and weight compared with the nontreated positive control (Group 1). A significant statistical difference was observed between all groups (*p* < 0.05). The difference between all treatment groups among themselves was not significant (*p* > 0.05) (Table 1). TH and MH dosages showed comparable results for tumour multiplicity, but TH showed a higher % reduction in size of primary tumours than MH. TH presented a lower tumour size and weight when compared with MH (Table 1).

### 3.3. Tumour Progression

**Figure 1:**
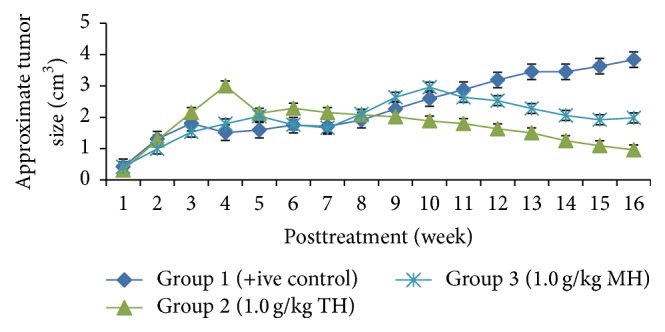
The progression of tumor size (cm^3^) in TH and MH treated groups compared with the nontreated positive control. Data is presented as mean ± SEM. A mixed model two-way repeated measures ANOVA (*p* < 0.05 in all weeks). TH: Tualang honey, MH: Manuka honey, and +ive control: group bearing breast cancer but received no honey treatment.

The size of the tumours measured weekly showed that the tumours in treated groups (Groups 2 and 3) had a slower size increment with a lesser median tumour size of less than ≤3 and ≤2.96 cm^3^ for TH and MH, respectively, during the whole experimental period of 16 weeks. Tumours in the control group showed a rapid progression over period of time reaching up to 3.84 cm^3^ in size. The statistical difference was not significant for the tumour size progression between treatment groups and control (*p* > 0.05); however, a significant difference was observed only in the last few weeks (*p* < 0.05) (Figure 1). The difference of tumour progression between all treatment groups among themselves was not statistically significant (*p* > 0.05). Few of the cancer masses in TH treated groups had regressed to nonpalpable state during the experimental period. TH showed a slower tumour progression compared with MH (Figure 1).

### 3.4. Body Weights

**Figure 2:**
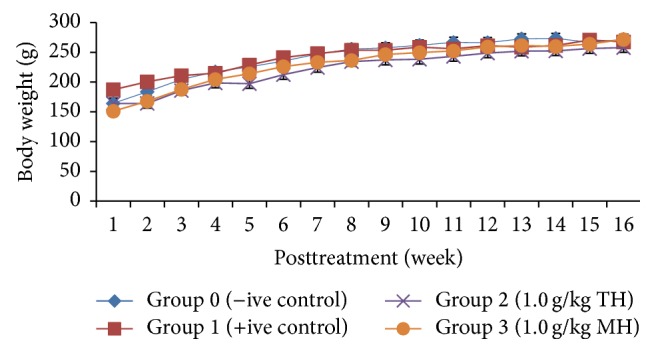
Body weight progression among all groups of rats. Data is presented as mean ± SEM and a mixed model two-way repeated measures ANOVA was conducted to analyze the results. A positive body weight progression was observed over time (*p* > 0.05). BW: body weight, ABW: actual body weight, TH: Tualang honey, MH: Manuka honey, −ive control: normal rats, and +ive control: group bearing breast cancer but received no honey treatment.

In general, body weights of the rats in all groups (treated groups & nontreated control groups) gradually increased throughout the experimental period over time (Figure 2). Data for median body weights of rats in each group is presented in Table 2. At week 16, all the rats in TH and MH treated groups showed a higher BW change% compared with the negative and positive controls. The difference in percentage body weight change (BW change%) between all groups was found statistically not significant (*p* > 0.05) (Table 2).

Further analysis on the weight gain was performed by evaluating the actual body weights of rats at week 16 by subtracting the total tumour weight from the body weight of the rats obtained at week 16. The difference in median actual body weight (ABW) of treated groups compared with negative and positive control groups was also not significant (*p* > 0.05). The rats in TH and MH treated groups presented a higher median actual body weight compared with the rats of nontreated positive control. The rats in TH and MH treated groups also presented a significant higher percentage of change in actual body weight gain (ABW change%) than the rats in nontreated positive control. The difference between all treatment groups among themselves was not statistically significant (*p* > 0.05). Overall, treatment with TH and MH showed a positive effect on actual body weight gain (after subtraction of tumours weight) compared with the nontreated positive control. MH presented a higher actual body weight gain than a similar dose of TH (Table 2).

### 3.5. Macroscopic Evaluation of the Tumours

The tumour masses in the nontreated control (Group 1) were found solid, larger in size, and hard in consistency, exhibiting areas of necrosis and hemorrhage. Some of these tumours exhibited pus-like material (necrotic tissue), exuding from the tumours when sectioned. TH and MH treated groups (Groups 2 and 3) had tumours which were softer, paler, and smaller in size.  Table 4 shows the effect of TH and MH treatment on gross appearance, size, and texture of tumours compared with the nontreated control.

### 3.6. Tumour Grading and Histological Features

This was concluded from the grading results that the majority of the tumour specimens in the control group showed patterns of grade III compared with the groups treated with TH and MH (Groups 2 and 3) which exhibit tumours mainly of grade I and II. Data of the histological grading for the cancer specimens are presented in Table 3. In all groups, the majority of the tumours were found to be adenocarcinomas. Tumours in the control group were observed to have increased heterogeneous nuclei formation, which were hyperchromatic, vesicular, and highly pleomorphic, with moderate cytoplasm and increased mitotic activity compared with TH and MH treated groups which had fatty tissue with small nucleus and cystic spaces (Table 4). TH treated groups presented a higher percentage of grade I tumours compared with those treated with MH. Major types of carcinoma identified in all groups were benign, DCIS (ductal carcinoma in situ), micropapillary, and NOS (not-otherwise specified) (Table 6). The majority of the specimens in the positive control group showed NOS types of carcinoma (40/49, 81.63%), followed by micropapillary (4/49, 8.16%), DCIS (3/49, 6.12%), and benign (2/49, 4.08%). In group treated with 1.0 g/kg TH, 48.38% carcinomas (15/31) were of types NOS followed by benign (8/31, 25.80%) and micropapillary (8/31, 25.80%) types. The group treated with MH 1.0 g/kg had NOS (25/35, 71.42%) as the major type of carcinoma followed by micropapillary (8/35, 22.85%) and benign (2/35, 5.71%) (Table 5). The percentage of benign patterns was found higher in TH and MH treated groups compared with the nontreated control. In summary, the cancers which developed in TH and MH treated groups had less aggressive tumours behavior compared with cancers developed in nontreated control.

### 3.7. Haematological Parameters

The results of haematological parameters of negative control were used to establish a normal or standard reference range. Treatment with TH and MH showed a potentiating effect on Hb, RBC, PCV, lymphocytes, and eosinophils compared with the nontreated positive control, while, these honeys showed a lowering effect on polymorphs and monocytes. It was also observed that MH showed a slightly potentiating effect, and TH showed a lowering effect on TWBC when compared with the nontreated positive control. MH demonstrated a slightly potentiating effect on MCV compared with the nontreated positive control. However, TH showed no significant potentiating or lowering effect on MCV compared with the nonpositive control. It was found that the level of MCH and MCHC was almost comparable for TH and MH compared with the nontreated positive control. The results for RDW showed that TH and MH treatments showed a slightly lowering effect on RDW compared with the nontreated positive control. Treatment with TH showed a potentiating effect on platelets count, while, MH presented a lowering effect compared with the nontreated positive control. The difference for potentiating or lowering effect on different parameters was minute between TH and MH themselves. The detailed results with statistical analyses are presented in Table 7.

### 3.8. Determination of Serum Level Concentration of Apaf-1, IFN-*γ*, TNF-*α*, and E2

**Figure 3:**
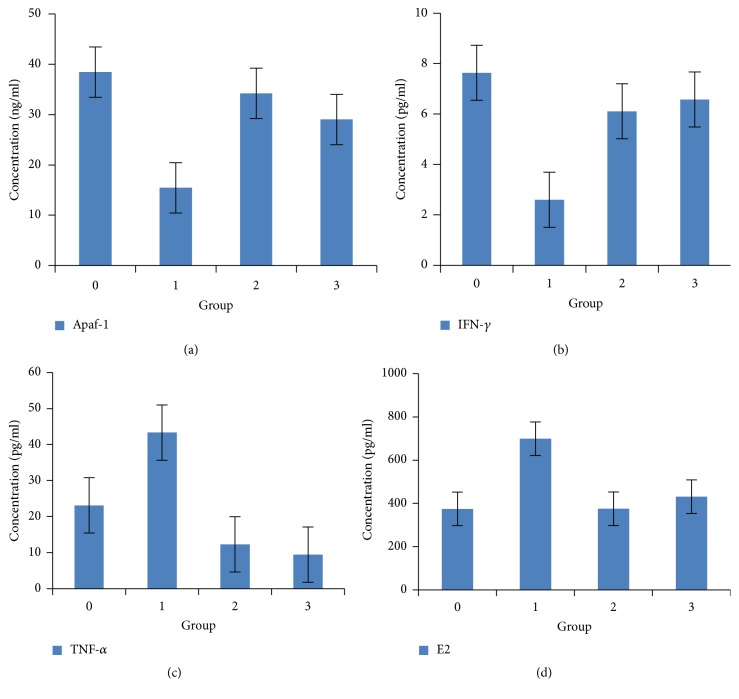
The serum level concentration of Apaf-1 (ng/ml), IFN-*γ* (pg/ml), TNF-*α* (pg/ml), and E2 (pg/ml) in the rats of TH and MH treated groups compared to the rats of negative and positive controls. Group 0: negative control (normal rats), Group 1: positive control, Group 2: 1.0 g/kg TH, and Group 3: 1.0 g/kg MH. Data are expressed as median interquartile range (IqR) using Kruskal-Wallis test. Values are statistically significant, *p* < 0.05. Apaf-1: apoptotic protease activating factor 1, IFN-*γ*: interferon gamma, TNF-*α*: tumour necrosis factor alpha, E2: estradiol, TH: Tualang honey, and MH: Manuka honey.

Serum levels of Apaf-1, IFN-*γ*, TNF-*α*, and E2 in the negative control group (Group 0) were used to establish a normal reference range. The rats treated with TH and MH (Groups 2 and 3) showed a higher median concentration of Apaf-1 and IFN-*γ* but a lower TNF-*α* and E2 concentration compared with the those of nontreated positive control (Figure 3). A significant statistical difference was observed between all groups (*p* < 0.05). The difference between all treatment groups among themselves was not significant (*p* > 0.05). TH presented a slightly higher Apaf-1 concentration and comparable results for IFN-*γ* when compared to a similar dose of MH. TH showed a lower E2 concentration and a slightly higher TNF-*α* concentration compared to MH (Figure 3).

### 3.9. Expression of Apaf-1, Caspase-9, p53, FASLG, FADD, IFNGR, ESR1, COX-2, Bcl-xL, and TNF-*α* in Tumour Specimens

The tumour specimens of TH and MH treated groups showed a higher % expression or positivity of Apaf-1, Caspase-9, p53, and IFNGR1 yet a lower % expression of ESR1, COX-2, Bcl-xL, and TNF-*α* compared to those of control (Table 8). A higher percentage of immunopositive cells was also observed in TH and MH treated tumours compared to the tumours of nontreated control (Tables 9 and 10). A significant statistical difference was observed between different groups (*p* < 0.05). The difference between all treatment groups among themselves was not significant (*p* > 0.05). TH showed a slightly higher % expression of Apaf-1 and a lower expression of p53 and IFNGR1 compared to a similar dose of MH. TH and MH showed almost comparable results for the % expression of Caspase-9. While comparing with MH, TH showed a slightly higher % expression of ESR1, COX-2, and Bcl-xL and comparable results for TNF-*α* (Table 8). Tumours treated with TH and MH showed no expression of FASLG and FADD (0% expression or positivity). A very minute expression of these proteins was observed in tumour specimens of nontreated positive control (Tables 8 and 9).

## 4. Discussion

Chemotherapy is one of the most commonly used treatment modalities against cancer. A major concern for anticancer chemotherapeutic drugs is their potential toxicity [3]. The existing therapeutic drugs can be complemented by alternative medicine [5]. Published data show that honey has antitumour [17], antiproliferative [17], antineoplastic [30], and antimutagenic [31] effects. Malaysian TH and Australian/New Zealand MH have been shown to have anticancer effects [14, 17]. Our study demonstrates some novel mechanistic findings regarding the utility of TH and MH as potential therapeutic agents against breast cancer. Our study showed that the treatment using the same strengths of TH and MH as a therapy was highly effective to reduce the size of primary tumours. The rats that received TH and MH showed a slower tumour progression and lower multiplicity, size, and weight compared with those of nontreated positive control (Table 1). Such changes were also confirmed on gross macroscopic evaluation (Table 4). Treatment with TH and MH seems to be capable of reversing the tumourigenesis, which is evident by the reduction in tumour size and weight in treated groups. This modulatory effect of TH and MH on tumour progression, multiplicity, size, and weight could be attributed to strong antioxidant [9] and antimutagenic [31] activity of honey. Carcinogenesis is a multistep process. It can be divided into three main stages, initiation, promotion, and progression [32]. Cancer-therapeutic agents may act as antipromoting agents via intervening at initiation or promotion stages of carcinogenesis [32]. Thus, we can assume that TH and MH may intervene at the initiation or promotion stage to inhibit tumour progression. This is the reason that the tumours treated with honey appeared smaller in our study. The lower tumour multiplicity shows that TH and MH act as antimetastatic agent. Some of the breast lesions in our study were found to be completely vanished at the end of the study. It has been demonstrated that tumours can be eliminated or diminished by chronic administration of low doses of chemotherapeutic drugs [33]. It can be hypothesized that TH and MH treatments behaved similarly. A study by Kadir and colleagues reported that honey may modulate tumour multiplicity, size, weight, and progression [30]. That study examined the cancer-preventive effects of TH using a different carcinogen DMBA (7,12-dimethylbenzanthracene), while we conducted a cancer-therapeutic study using the carcinogen MNU which has several advantages of organ specificity and tumours of ductal origin compared to DMBA [26]. Another study has also shown antitumoural effects of honey in animal models [34]. It has been reported that polyphenols, phenolic acids, and flavonoids are solely responsible for the antitumoural effects of honey [35]. The prominent profile of these compounds includes benzoic acid, gallic acid, syringic acid, p-coumaric acid, hyacinthin, trans-cinnamic acid, caffeic acid, salicylic acid, 3-phenyllactic acid, 3,5-dimethoxybenzoic acid, catechin, naringenin, luteolin, apigenin, desoxyanisoin, pinocembrin, chrysin, and tectochrysin [8]. Thus, antineoplastic or antitumoural effects of TH and MH may also be attributed to these compounds.

Assessment of histological features of the cancers has pivotal importance for the prognosis to determine the appropriate treatment [36]. Our study showed that the cancer masses in TH treated groups were in grade I and II or less aggressive compared with the control which had majority of grade III (Tables 3 and 4). It was observed that the tumours in the control group were highly pleomorphic with hyperchromatic nuclei, moderate cytoplasm arranged in sheets or nests, and acinar structures with increased mitotic counts. While TH and MH treated tumours showed low to moderate nuclear pleomorphism, fatty tissue with small lobules and moderate cytoplasm were likely to acquire normal breast structure. Honey is antimutagenic [31]; the better grade of the cancers in TH treated groups is probably due to its antimutagenic effects. Our study demonstrates that tumours of benign type were found more frequently in treatment groups compared with the nontreated control. The majority of the breast tumours in our study of MNU-induced model were invasive ductal carcinoma, and the commonest were of DCIS, micropapillary, and NOS (Tables 5 and 6). Thus, TH and MH seem to act at cellular level by reducing heterogeneous nuclei formation and mitotic activity to improve histological grading and patterns in tumours. This could probably lead to less aggressive types of tumours in treated groups compared with the tumours of nontreated control with more aggressive patterns.

The results of our study show that similar strengths of TH and MH showed a positive effect on body weight gain compared with the non-treated positive control (Table 2 and Figure 2). The reason positive control rats are not gaining as much weight as honey treated rats is perhaps due to cancer catabolism. Cancer is a catabolic state. Thus, cancer patients lose a lot of weight with worse outcomes [37]. It is hypothesized that TH and MH might be able to improve body weight gain.

It has been demonstrated that haematological parameters are correlated with prognosis of cancer [18]. Pre- and posttreatment studies have shown that breast cancer patients have abnormal or poor blood parameters [18]. We observed intriguing findings of blood parameters after the administration of TH and MH in the rats bearing breast cancer. Treatment with similar strengths of TH and MH had a potentiating effect on the haematological parameters such as RBC, Hb, PCV, lymphocytes, and eosinophils. These treatments presented a slight lowering effect on the levels of RDW, polymorphs, and monocytes compared to the nontreated positive control (Table 7). Research has reported a lower level of RBC, Hb, MCV, MCH, MCHC, and lymphocytes in pre- and posttreatment breast cancer patients. Anaemia was also reported in these patients due to iron deficiency [18, 38]. A higher level of RDW, TWBC, and polymorphs can be observed in breast cancer patients [18]. There are studies which have reported conflicting results on platelets count in pretreatment breast cancer patients [18, 38]. A study reported no relationship between curability of cancer and basophils, and eosinophils, and monocytes [18]. Our findings show that TH and MH may alter or tend to normalize these parameters to ameliorate carcinogenesis in breast cancer. The findings of our study also suggest that honey may modify Hb, RBC, and PCV to ameliorate anaemia. Exclusive honey feeding in the absence of any disease significantly modifies the haematological parameters [39].

Apaf-1 is reported as an essential target in the intrinsic apoptotic pathway to regulate Caspase-9 pathway apoptosis [20]. Its loss results in tumour cells evading apoptosis [20]. Our data reported that TH and MH cause increase of the concentration of Apaf-1 at serum level (Table 4). The finding is validated when we observed a higher expression of this protein in treated cancer masses as well (Figure 3). Similarly, TH and MH were found to potentiate the expression of proapoptotic proteins Caspase-9 and p53. We may postulate that TH and MH caused upregulation of the expression of Apaf-1, Caspase-9, and p53 and thus may activate the intrinsic apoptotic pathway to promote apoptosis. It is evidenced by regressed growth patterns and the low histological grading in treated tumours. The possible mechanism demonstrates that TH and MH akin to chemotherapeutic agents may induce apoptosis through multiple signaling pathways that converge on the mitochondria to cause the release of cytochrome c. Cytochrome c binds to Apaf-1 in the presence of dATP/ATP (deoxyadenosine triphosphate/adenosine triphosphate), which then binds to procaspase-9 to form a cytochrome c–Apaf-1–Caspase-9 complex, called apoptosome. Apoptosome enables enzymatic self-activation of Caspase-9 that subsequently activates procaspase-3. This ultimately results in cell death [20]. Honey mediates apoptosis mainly through the intrinsic apoptotic pathway and by enhancing proapoptotic proteins expression [5, 14]. p53 in the intrinsic apoptotic pathway exhibits Apaf-1 as an essential downstream target to regulate apoptosis [20]. A similar finding reports that honey causes increase in p53 expression in colon cancer in its anticanner effect [40]. We can assume that TH and MH act as therapeutic agents against breast cancer by modulating p53 expression through the intrinsic apoptotic pathway with the involvement of Apaf-1 and Caspase-9.

Our findings showed no evidence of the activation of FASLG and FADD, hence no involvement of Caspase-8 or the extrinsic apoptotic pathway in TH and MH mediated apoptosis. Our results are in line with another study which demonstrated that Manuka honey induces intrinsic or Caspase-9 apoptotic pathway in breast cancer. This study showed no evidence of the involvement of Caspase-8 pathway [17].

Investigations on human tumour cells have reported that the resistance of a variety of tumour cell lines to IFN-*γ* is owing to the defects in IFNGR-mediated signaling cascades [41]. The reduced expression of IFNGR1 is tightly associated with poor prognosis and more aggressiveness of cancer [41]. This emphasizes the importance of the expression of IFNGR1 in the prevention of cancer. The higher expression of IFNGR in TH and MH treated cancer tissues is supported by higher expression of IFN-*γ* at serum level in our treated groups. Thus, TH and MH treatments cause a higher expression of IFN-*γ* as well as its binding receptor IFNGR1. The results of our study are supported by a research reporting that honey may cause high serum levels of IFN-*γ* which inhibit tumour formation [42]. Our study provides evidence that Tualang and Manuka honeys are novel immune potentiators which induce IFN-*γ* and IFNGR1 expression to potentiate IFN-*γ* activities in breast tumour cells.

TNF-*α* has been shown to play a dual role, beneficial and deleterious effect for the promotion or inhibition of infectious diseases [43]. Investigations strongly suggest that the expression of TNF-*α* in breast tumours actually promotes tumour growth. The higher expression of TNF-*α* in inflammatory breast carcinoma was found to be associated with increasing tumour grade and the metastatic behavior of breast carcinomas [23]. Our study shows a lower TNF-*α* concentration at serum level as well as a lower expression in tumour specimens of treated groups, which further validates our results. TNF-*α* is produced by monocytes [44], and our study also shows that TH and MH treatments cause lowering of the monocytes level in blood. This validates that TH and MH hinder this signaling pathway by lowering TNF-*α* as well as monocytes to cause the anticancer effects. Research has shown that MH stimulates monocytes to release tumour necrosis factor-alpha [45] to enhance immune response. But, this study has reported modulatory effect of honey on TNF-*α* in disease-free models, contrary to cancer models of our study. We can postulate that TH and MH are anti-TNF-*α* agents.

A proinflammatory marker,* COX-2*, is overexpressed in breast cancer and might be a crucial therapeutic target in breast cancer. It leads to the destruction of the basal membrane and the formation of new blood vessels allowing tumour growth [24]. Our study suggests that TH and MH treatment may inhibit COX-2 pathway cell proliferation-related transcriptional programs in breast carcinomas. This mechanism is validated by the reduced tumour size in our treated groups. Honey has been reported to be involved in regulation of COX-2, as an anti-inflammatory agent [46]. Phenolic acids and polyphenols in honey have been reported to be responsible for anti-inflammatory activity induced by COX-2 pathway [46]. It can also be hypothesized that COX-2 inhibition by TH and MH may cause lower tumour multiplicity through reduction in inflammation caused by COX-2. The proposed mechanism by which TH and MH may inhibit COX-2-induced inflammation in carcinogenesis is supported by previous findings [46]. These findings proposed that honey may inhibit inflammation through suppression of NF-*κ*B pathway by blocking this signaling pathway. Thus, it may be presumed that TH and MH may intervene in this inflammatory signaling pathway to downregulate the COX-2 expression. COX-2 expression is regulated by TNF-*α* and our study also shows a lower expression of TNF-*α*. This further validates the hindering of this inflammatory pathway by TH and MH.

A study has reported that overexpression of Bcl-xL in breast cancer patients is associated with metastasis and worse prognosis [25]. The decrease of Bcl-xL expression observed in TH and MH treated tumours suggests that the administration of TH and MH can lead to lower tumour cells proliferation and increased apoptosis. Bcl-xL exerts its effect through the loss of mitochondrial outer membrane integrity by blocking both mitochondrial swelling and membrane hyperpolarization [47]. Our findings suggest that Bcl-xL expression was hindered by TH and MH at its intrinsic mitochondrial apoptotic pathway. This ultimately promotes apoptosis through increased expression of mitochondrial pathway proteins, Caspase-9, p53, and Apaf-1, as observed in our study. A study on walker 256 breast carcinoma verified that honey may modulate the expression of pro- and antiapoptotic proteins in animal models [34].

The findings of our study show that TH and MH showed a lowering effect on E2 concentration at serum level (Figure 3) and ESR1 at cancer tissues level (Tables 8 and 10). Breast cancer patients had higher levels of estrogens and ER-mediated bioactivities [19]. Higher serum level of E2 has been reported in breast cancer patients [19]. It promotes cell proliferation and suppresses apoptosis by directly modulating the genes transcription. Thus, estrogen is considered as an important target in breast cancer treatment. It has been reported that treatment with estrogen-lowering drugs shrinks tumours of breast cancer patients [48]. Thus, TH and MH act as natural estrogen-lowering drugs and shrink tumour size as reported in our study. The inhibition of ESR1 may decrease the risk for hormonal breast cancer [19]. Research has shown that exogenous or synthetic estradiol (E2) can be used as a treatment in ER positive breast cancer to cope with ER proliferative pathway and to stimulate the apoptotic pathway [49]. It is also possible that honey, which is a natural phytoestrogen [7], plays its role in modulating the endogenous estrogen and estrogen receptors and may stimulate the apoptotic pathway. Based on our results, we can hypothesize that TH and MH may reduce E2 and ESR1 expression to inhibit tumour growth. TH and MH may bind to the ER, similar to drugs, and may disrupt receptor dimerization by inhibiting cytoplasmic-nuclear shuttling to block ER nuclear localization. It will bind to the ERs and therefore prevent its activation by estrogen, leading to its degradation similar to ERs targeting drugs. Honey exerts estrogen agonistic effect at high concentrations (20–100 *μ*g/mL) and antagonistic effect at low concentrations (0.2–5 *μ*g/mL) [50]. This antiestrogenic effect was attributed to its flavonoids or polyphenols content [50]. This study reported in vitro analysis of estrogen receptors only. Our study reports in vivo analysis of E2 and ESR1 with an antagonist effect of TH and MH. It was also observed that MH showed better effects in terms of tumour multiplicity, reduction, size and weight, and mildness in histological features. It also showed better results for some markers at haematological, serological, and cancer tissues level. This difference in the efficacy of two honeys may be attributed to their floral origin and compositional differences. Overall, TH and MH if tested for antibreast cancer potency, they are akin to manage the disease through modulation of different pathways involved, as reported in our results. Studies have elucidated various classes of active ingredients in honey which act synergistically to bring about the maximum therapeutic efficacy [5], making honey a novel anticancer agent of great interest.

## 5. Conclusions

Tualang and Manuka honeys exhibit antibreast cancer activity. The mechanism by which TH and MH exert cancer-therapeutic effects is multifold, through modulation of immune response by ameliorating haematological and serological parameters and by activation of the intrinsic apoptotic pathway through upregulation of the expression of proapoptotic proteins such as Caspase-9, Apfa-1, p53, IFN-*γ*, and IFNGR1. Concomitantly, these honeys downregulate the expression of antiapoptotic proteins such as Bcl-xL, TNF-*α*, COX-2, E2, and ESR1. We believe that current findings should facilitate further research to investigate whether TH and MH could synergize with or be a substitute for chemotherapeutic drugs given at suboptimal doses for cancer therapy. The most reliable study on the usefulness of TH and MH as natural cancer-therapeutic agent or adjuvant is to conduct research in more animal and clinical trials.

## Figures and Tables

**Table 1:** Tumour multiplicity, % reduction, size, and weight in TH and MH treated groups compared with the nontreated control.

Tumor	Groups			*p* value
	1 +ive control	2 (1.0 g/kg TH)	3 (1.0 g/kg MH)	
^*∗*^Multiplicity	5 (4)	3 (5.25)	3 (3.5)	0.462
^*∗*^% reduction	0 (0)	70.82 (22.94)	57 (32.94)	0.000
^*∗*^Size (cm^3^)	1.23 (2.49)	0.17 (0.29)	0.44 (1.11)	0.000
^*∗*^Weight (g)	2.55 (7.76)	0.89 (2.62)	1.8 (3.70)	0.011

^*∗*^Kruskal-Wallis test. Data are expressed as median interquartile range (IqR). Values are statistically significant when *p* ≤ 0.05. Multiplicity: number of tumours developed, % reduction: the percentage reduction in size of primary tumours, TH: Tualang honey, MH: Manuka honey, and +ive control: group bearing breast cancer but with no honey treatment.

**Table 2:** Body weight measurements of rats among all groups at week 1 and week 16.

Body weight	Groups				*p* value^a^
	0 −ive control	1 +ive control	2 (1.0 g/kg TH)	3 (1.0 g/kg MH)	
BW at week 1	167.5 (32.25)	191.5 (94.5)	138 (60.25)	142 (53.25)	0.300
BW at week 16	272 (32.25)	270.5 (31)	238 (55.75)	268 (31.75)	0.392
BW change (%)	66.54 (37.16)	32.14 (90.05)	68.21 (18.42)	109 (56.22)	0.182
ABW at week 16	272 (37.25)	245.49 (17.05)	236.76 (32.21)	249 (39.11)	0.07
ABW change (%)	66.54 (37.16)	26.52 (64.48)	66.89 (23.88)	78.09 (48.02)	0.110

^a^Kruskal-Wallis test. Data are expressed as median interquartile range (IqR). Values are statistically significant when *p* ≤ 0.05. Percentage body weight change or gain (BW change%) = [(FBW − IBW) × 100]/IBW.

Actual body weight = Body weight at week 16 − weight of tumours.

Percentage actual body weight change or gain (ABW change%) = [(ABW − IBW) × 100]/IBW.

BW: body weight, FBW = final body weight, IBW: initial body weight, and ABW: actual body weight.

**Table 3:** Grading of tumours in groups treated with TH and MH compared with the tumours of nontreated control.

Tumor	Groups		
	1 +ive control	2 (1.0 g/kg TH)	3 (1.0 g/kg MH)
Total number	47	23	33
^*∗*^Grade I (%)	6 (12.76)	14 (60.86)	22 (66.66)
^*∗*^Grade II (%)	15 (31.91)	5 (21.73)	9 (27.27)
^*∗*^Grade III (%)	26 (55.31)	4 (17.39)	2 (6.06)

^*∗*^Fisher Exact test: statistically significant difference between the groups, *p* < 0.05. TH: Tualang honey; MH: Manuka honey, and +ive control: group bearing breast cancer but received no honey treatment.

**Table 4:** The gross morphology and histology of the breast tumours of rats in TH and MH treated groups compared with the nontreated positive control. The H & E stained sections examined under light microscopy at ×400 magnification. The majority of tumours in nontreated control group were of grade III with increased heterogeneous nuclei formation and mitotic activity (plate (a), arrow) compared with the tumours in TH and MH treatment groups which were of grade I and II (less aggressive) (B): (a) = +ive control, (b) = 1.0 g/kg TH, and (c) = 1.0 g/kg MH. TH: Tualang honey, MH: Manuka honey, and +ive control: group bearing breast cancer but received no honey treatment.

Study groups →	Group 1 +ive control	Group 2 1.0 g/kg TH	Group 3 1.0 g/kg MH
(A) The gross appearance of tumours	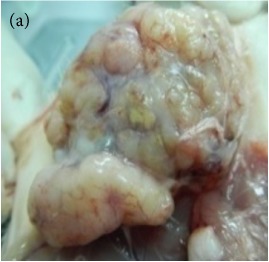	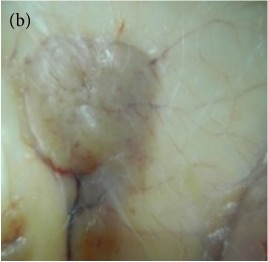	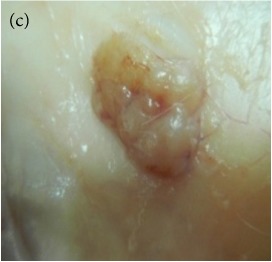
(B) The histology of the breast cancer	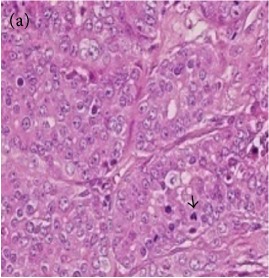	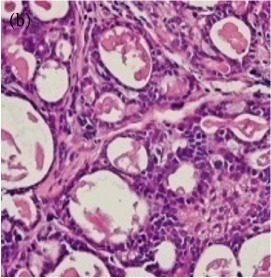	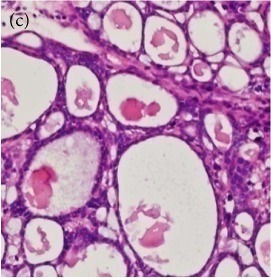

**Table 5:** The % age of histological patterns identified in TH and MH treated groups versus nontreated positive control.

Group	Total	Number of tumours (amount%)			
		Benign	DCIS	Micropapillary	NOS
1 +ive control	49	2 (4.08)	3 (6.12)	4 (8.16)	40 (81.63)
2 (1.0 g/kg TH)	31	8 (25.80)	0 (0)	8 (25.80)	15 (48.38)
3 (1.0 g/kg MH)	35	2 (5.71)	0	8 (22.85)	25 (71.42)

TH: Tualang honey, MH: Manuka honey, DCIS: ductal carcinoma in situ, NOS: not-otherwise specified, and +ive control: group bearing breast cancer but received no honey treatment.

**Table 6:** The histological patterns of tumours identified in breast cancer bearing rats among all groups. Cancers which developed in honey treated rats had less aggressive tumours behavior with more benign pattern compared with cancers developed in nontreated control; (a) benign, (b) DCIS, (c) micropapillary, and (d) NOS. DCIS: ductal carcinoma in situ and NOS: not-otherwise specified.

Magnification	Benign	DCIS	Micropapillary	NOS
×200	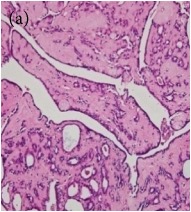	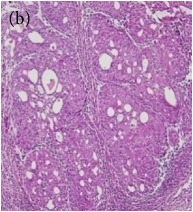	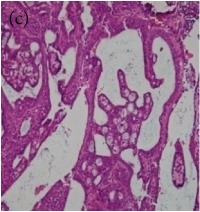	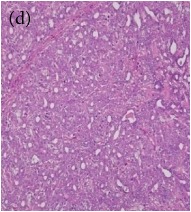
×400	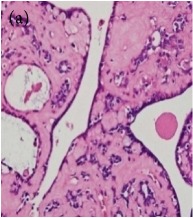	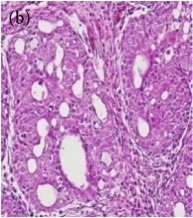	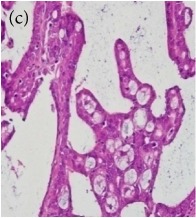	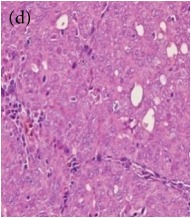

**Table 7:** The haematological parameters of TH and MH treated groups compared with negative and positive controls.

	Groups				*p* value^a^
	1 −ive control	2 +ive control	3 (1.0 g/kg TH)	4 (1.0 g/kg MH)	
RBC (10^12^/L)	7.35 (0.42)	5.1 (0.9)	6.8 (3.32)	6.15 (2.75)	0.003
Hb (g/dl)	15.2 (0.77)	11.35 (1.42)	13.85 (5.95)	13.85 (4.45)	0.003
PCV (%)	46 (3.25)	35 (8.25)	42.5 (17.75)	43.5 (12.25)	0.009
MCV (fl)	68.5 (3.25)	65 (4.75)	65 (11.75)	67 (10.25)	0.013
MCH (pg)	20.5 (1)	21 (2)	21 (3)	21 (3.5)	0.169
MCHC (g/L)	32 (1)	31.5 (2.25)	32 (3.5)	31.5 (2.25)	0.062
RDW (%)	11.9 (1.57)	13.95 (1.72)	12.25 (2.17)	12.65 (2.1)	0.01
TWBC (10^9^/L)	4.75 (1.75)	6.4 (7.52)	4.82 (8.75)	7.35 (6.85)	0.02
Polymorphs (%)	32 (8.75)	46.5 (18)	31.5 (11.25)	31.5 (9.5)	0.01
Lymphocytes (%)	68 (8)	49 (19.25)	69 (9.75)	67.5 (4.5)	0.014
Monocytes (%)	2 (1.5)	2.5 (3.5)	0.5 (1)	1 (4.25)	0.231
Eosinophils (%)	0 (1)	0 (1.25)	0.5 (1)	1 (0.25)	0.102
Basophils (%)	0	0	0	0	1
Platelets' (10^9^/L)	839 (225.75)	627.5 (196.75)	666.5 (229.25)	540.5 (324.75)	0.01

^a^Kruskal-Wallis test. Data are expressed as median interquartile range (IqR). Values are statistically significant when *p* ≤ 0.05. FBC: full blood count, RBC: red blood cells, Hb: haemoglobin, PCV: packed cell volume, MCV: mean corpuscular volume, MCH: mean corpuscular haemoglobin, MCHC: mean corpuscular haemoglobin concentration, RDW: red cell distribution width, TH: Tualang honey, MH: Manuka honey, −ive control: normal rats, and +ive control: group bearing breast cancer but received no honey treatment.

**Table 8:** The immunohistochemical expression of pro- and antiapoptotic proteins in tumours treated with TH and MH compared with the tumours of nontreated control.

Tumors	Groups		
	1 +ive control	2 (1.0 g/kg TH)	3 (2.0 g/kg MH)
Total number	40	23	30
Number of Caspase-9 positive tumors (% expression)	12 (30)	16 (69.56)	21 (70)
Number of Apaf-1 positive tumors (% expression)	15 (37.5)	15 (65.21)	19 (63.33)
Number of p53 positive tumors (% expression)	17 (42.5)	14 (60.86)	20 (66.66)
Number of FASLG positive tumors (% expression)	15 (37.5)	0	0
Number of FADD positive tumors (% expression)	13 (32.5)	0	0
Number of IFNGR1 positive tumors (% expression)	20 (50)	17 (73.91)	25 (83.33)
Number of TNF-*α* positive tumors (% expression)	30 (75)	17 (73.91)	22 (73.33)
Number of COX-2 positive tumors (% expression)	26 (65)	11 (47.82)	13 (43.33)
Number of ESR1 positive tumors (% expression)	32 (80)	14 (60.86)	17 (56.66)
Number of Bcl-xL positive tumors (% expression)	31 (77.5)	11 (47.82)	13 (43.33)

Kruskal-Wallis test; statistically significant differences between the groups, *p* < 0.05.

FASLG: fas ligand, FADD: fas-associated via death domain, IFNGR1: interferon gamma receptor 1, TNF-*α*: tumour necrosis factor alpha, COX-2: cyclooxygenase-2, ESR1: estrogen receptor 1, Bcl-xL: B-cell lymphoma extra large, +ive control: group bearing breast cancer but received no treatment, TH: Tualang honey, and MH: Manuka honey.

**Table 9:** The immunohistochemical expression of proapoptotic proteins in TH and MH treated tumours compared with the tumours of nontreated control; (a) +ive control for immunohistochemistry (IHC) analysis, (b) +ive control for study (group bearing breast cancer but received no honey treatment), (c) 1.0 g/kg TH, and (d) 1.0 g/kg MH. All specimens were examined at ×400 microscopic magnification and brown color showed antibody positivity. FASLG and FADD showed no expression in tumours of all treatment groups, while tumours in treated groups showed a higher expression of proapoptotic proteins than those of nontreated control. FASLG: fas ligand, FADD: fas-associated via death domain, IFNGR1: interferon gamma receptor 1, TH: Tualang honey, and MH: Manuka honey.

Proteins	+ive control (for IHC)	Group 1 +ive control	Group 2 1.0 g/kg TH	Group 3 1.0 g/kg MH
Caspase-9, IHC +ive control is human colon	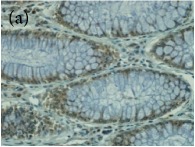	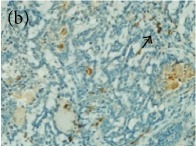	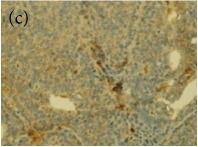	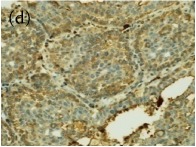
Apaf-1, IHC +ive control is human colon	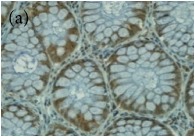	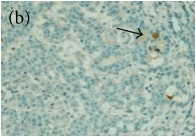	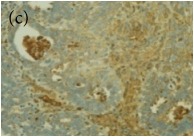	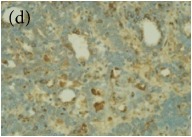
P53, IHC +ive control is human breast cancer	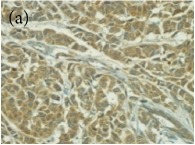	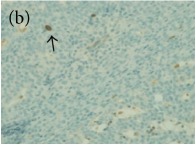	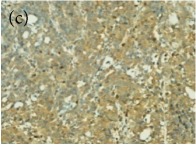	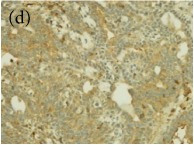
FASLG, IHC +ive control is human colon cancer	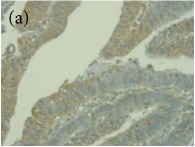	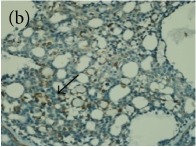	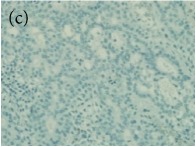	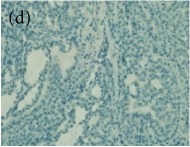
FADD, IHC +ive control is human breast cancer	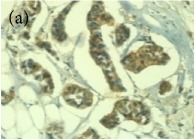	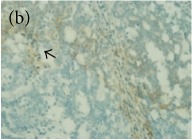	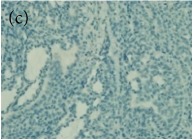	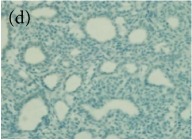
IFNGR1, IHC +ive control is human colon	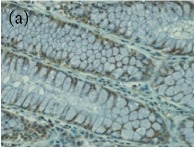	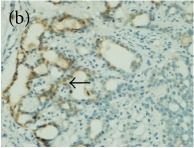	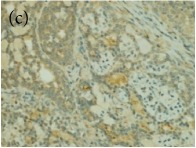	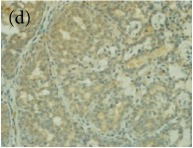

**Table 10:** The immunohistochemical expression of antiapoptotic proteins in TH and MH treated tumours compared with the tumours of nontreated control; (a) +ive control for IHC analysis, (b) +ive control for study (group bearing breast cancer but received no honey treatment), (c) 1.0 g/kg TH, and (d) 1.0 g/kg MH. All specimens were examined at ×400 microscopic magnification and brown color showed antibody positivity. Arrows in plates (c) and (d) show lower expression of antiapoptotic proteins in treated tumours compared with the nontreated control. TNF-*α*: tumour necrosis factor alpha, COX-2: cyclooxygenase-2, ESR1: estrogen receptor 1, Bcl-xL: B-cell lymphoma extra large, TH: Tualang honey, and MH: Manuka honey.

Proteins	+ive control (for IHC)	Group 1 +ive control	Group 2 1.0 g/kg TH	Group 3 1.0 g/kg MH
TNF-*α*, IHC +ive control is human breast cancer	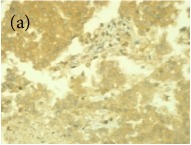	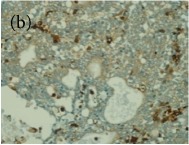	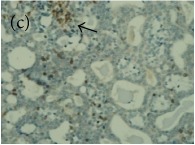	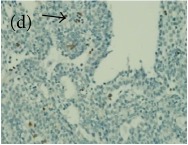
COX-2, IHC +ive control is human colon cancer	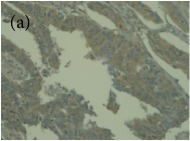	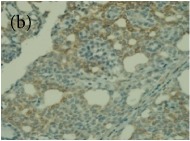	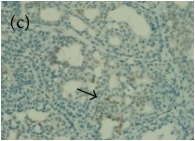	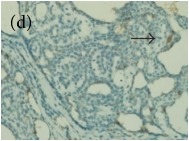
ESR1, IHC +ive control is human breast cancer	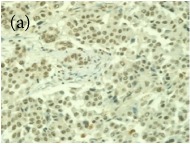	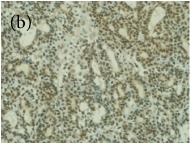	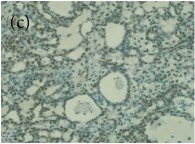	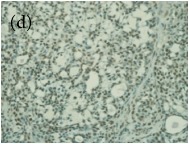
Bcl-xL, IHC +ive control is human tonsils